# A novel and unique refraction-based optical recording system for pharmacological investigations on isolated muscle preparations

**DOI:** 10.1016/j.mex.2023.102117

**Published:** 2023-03-17

**Authors:** Zsolt Pirger, Zita László, Ildikó Kemenes, György Kemenes, István Fodor

**Affiliations:** a Ecophysiological and Environmental Toxicological Research Group, Balaton Limnological Research Institute, Eötvös Loránd Research Network, Tihany, Hungary; b Sussex Neuroscience, School of Life Sciences, University of Sussex, Brighton, BN1 9QG, UK

**Keywords:** Pharmacology, Optical recording system, Isolated heart preparation, The great pond snail, *Lymnaea stagnalis*, In vitro pharmacological method based on a rapid, easy, cheap, and effective procedure and isolated heart preparations

## Abstract

In the field of neuroscience and ecotoxicology, there is a great need for investigating the effect(s) of a variety of different chemicals (e.g., pharmacologically active compounds, pesticides, neurotransmitters, modulators) at different biological levels. Different contractile tissue preparations have provided excellent model systems for *in vitro* pharmacological experiments for a long time. However, such investigations usually apply mechanical force transducer-based approaches. Thus, a rapid, easy, cheap, digital, and reproducible *in vitro* pharmacological method based on an effective, ‘non-invasive’ (compared to the force-transducer approaches), refraction-based optical recording approach and isolated heart preparations was developed.

• A versatile and unique refraction-based optical recording system with a Java application was developed.
• The recording system was tested and validated on isolated heart preparations obtained from the widely used invertebrate model organism, the great pond snail (*Lymnaea stagnalis*).
• The recording system illustrates the progression of technology from the mechanical force transducer system and can represent a suitable tool in ecotoxicology or neuroscience.

## Graphical abstract

**Figure:**
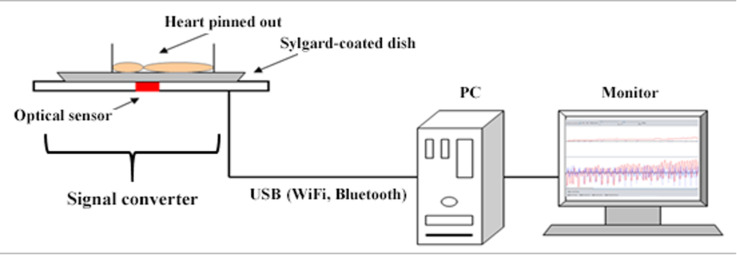

Specifications table

**Table:** 

Subject area:	Pharmacology, Toxicology and Pharmaceutical Science
More specific subject area:	Physiology
Method name:	*In vitro* pharmacological method based on a rapid, easy, cheap, and effective procedure and isolated heart preparations
Name and reference of original method:	N.A.
Resource availability:	Software availability: https://www.blki.hu/en/research#digirec

## Background

In the field of neuroscience and ecotoxicology, there is a great need to investigate the effects of chemicals at different levels. Since the 1970s, the great pond snail (*Lymnaea stagnalis*) has been a widely used invertebrate model in ecotoxicology for testing the effects of heavy metals, pesticides, and pharmacologically active compounds (reviewed by [1,^9^,12]) as well as in neuroscience for studying the functioning of the nervous system including chemical modulation (reviewed by [2,^5^,^9^,^11^,^13^,14].

The heart of *L. stagnalis* is an excellent organ for carrying out pharmacological experiments, including the study of (neuro)chemical modulation (reviewed by [2]). It can be said that such investigations on isolated heart preparations, not only in the case of *L. stagnalis* but also in general, utilize classical mechanical force transducer-based approaches. The aim of the present work was to develop a rapid, easy, cheap, digital, and *in vitro* pharmacological method based on an effective and non-invasive refraction-based optical recording approach and isolated heart preparations.

## Method details

### Materials and reagents

For preparing the Sylgard-coated dishes, Sylgard was purchased from Farnell (Sylgard 184 silicone elastomer kit; #101697) and prepared according to the manufacturers’ instructions. The *Lymnaea* physiological saline (pH = 7.5) for maintaining the isolated heart preparations contained 50 mM NaCl (#27788; VWR), 2 mM KCl (#26752; VWR), 4 mM CaCl_2_ (#22317; VWR), 4 mM MgCl_2_ (#J364; VWR), 10 mM TRIS (#93350, Merck). The physiological saline can be altered as desired according to the type of invertebrate or vertebrate tissue to be examined. For pharmacological experiments and method validation, dopamine analytical standard was purchased from Merck (#H8502) and a 10 µM solution was made in *Lymnaea* physiological saline.

### Heart preparations and animals

**Fig. 1:**
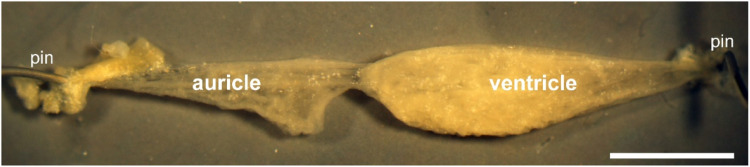
Dissected heart from *L. stagnalis* showing the auricle and ventricle. Scale bar = 2 mm.

For this study, isolated heart preparations (Fig. 1) were obtained from five-month-old *L. stagnalis* specimens which has been cultured in our laboratories in Brighton and Tihany. The animals were maintained in large plastic holding tanks (stocking density: 100 individuals/tank) containing 10L oxygenated artificial snail water with low copper content at a constant temperature of 20°C (±1°C) on a light:dark regime of 12 h:12 h. Snails were fed on lettuce ad libitum three times a week.

The physiology of the two-chambered heart of *L. stagnalis* is well-known: while heartbeat is generated by a muscle pacemaker located in the heart (i.e. myogenic), its regulation is basically implemented by many neurotransmitters and neuropeptides (e.g., serotonin, dopamine, and FMRFa) which are released by several types of motoneurons into the heart [3,^4^,[6], [7], [8],[17], [18], [19], [20].

**Fig. 2:**
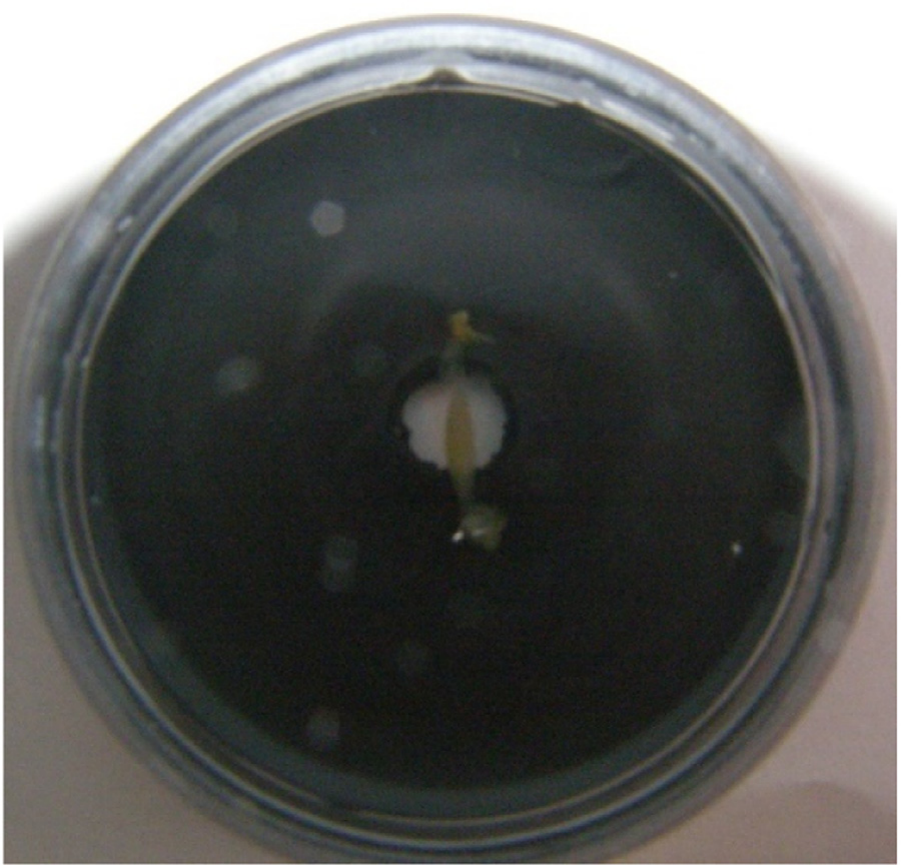
A pinned heart prepared from *L. stagnalis* on a Sylgard-coated dish. The hole in the Sylgard coat is for ensuring the way of the illumination of the optical sensor.

Individual hearts were dissected from the animals and individually pinned out without cannulation (i.e. both auricle and ventricle could contract) on Sylgard-coated dishes (Fig. 2) containing *Lymnaea* physiological saline. Pinning out serves a fundamental purpose, as the action of stretching the hearts stimulates the myogenic heartbeat [10,^15^,16]. Using a three-way tap (the perfusates can be switched quickly with only minimum mixing), pressure heads, and vacuum for wash out, the heart preparations can be easily maintained and perfused with physiological saline or the test chemical(s) in the dish.

### Refraction-based optical recording system

The optical recording system developed in our study is a novel and extremely unique method by which sensitive recordings of the heart muscle (or any contractile tissue) contractions can be made and subsequently analysed. In contrast to the classic mechanical force transducer set-up (muscle is fixed to a transducer, contractions are recorded and converted to digital format by an analogue-to-digital converter, amplified, displayed on an oscilloscope and/or computer, and analysed using a specific software), our system provides a rapid, easy, cheap, digital and ‘non-invasive’ recording.

**Fig. 3:**
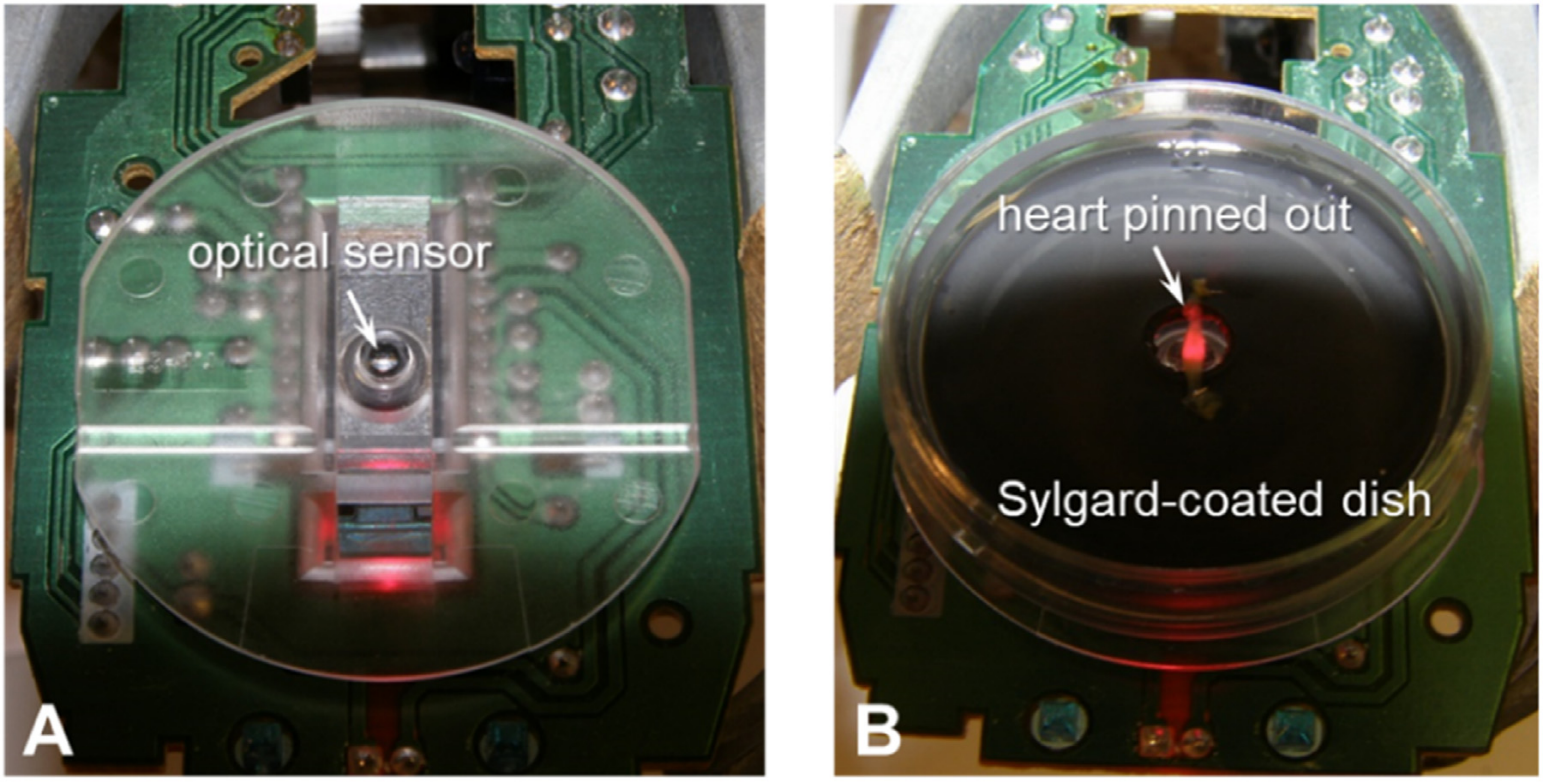
The optical signal converter without (**A**) and with the heart preparation (**B**).

The system utilizes the inbuilt optical sensor and signal converter of an optical/laser computer mouse that sensitively measures motions of the mouse dots per inch (DPI). This information is transmitted into a computer through a USB port (or even via WiFi or Bluetooth connection) and subsequently converted into cursor movements (graphs) on the monitor through a specialised software named DigiRec (detailed in the next subsection). In the present study, the optical sensor and the converter were taken out of the mouse (1600 DPI, 0.02 mm resolution) to be used for recording the movements made by the contracting heart. Importantly, the greater the DPI of the mouse applied, the greater the sensitivity. A heart preparation pinned out along either an X or Y axis is placed directly above the optical sensor (Fig. 3).

### DiciRec

The DigiRec application used for the recording was written in Java programming language and can be run under the Linux operation system. The program monitors the changes of the refraction caused by muscle contraction above the optical sensor and not the movements of the cursor on the screen, allowing a more accurate recording. The individual records are stored in the own file format of the software but the data can be exported in different well-known file formats (CSV, XLS) for further data processing.

**Fig. 4:**
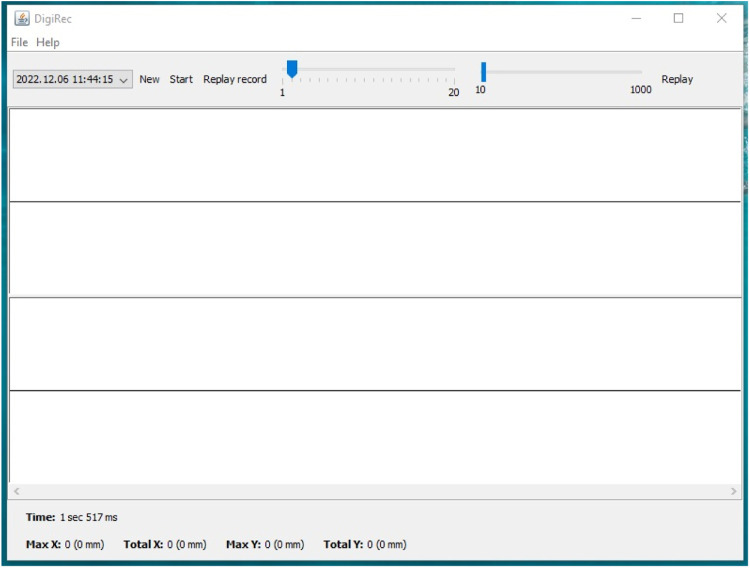
The graphical interface of DigiRec.

**Fig. 5:**
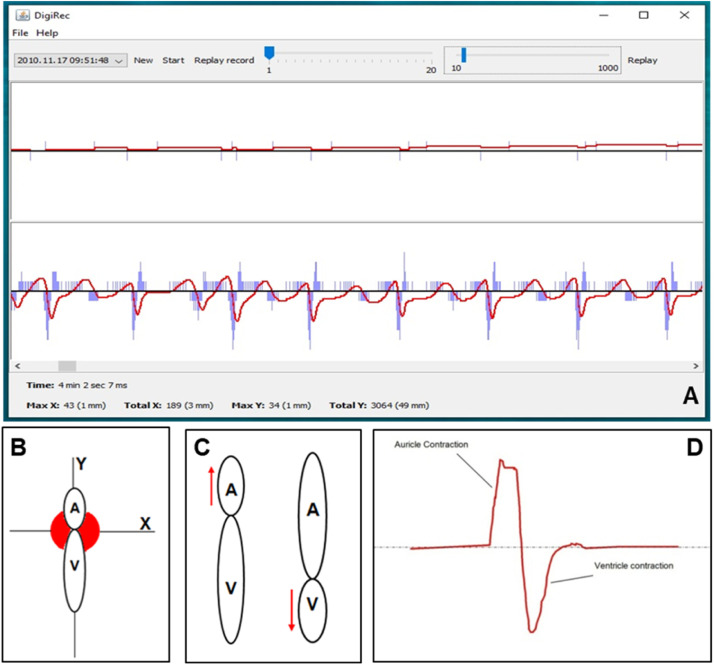
A representative record from an isolated heart preparation (**A**). The lower panel of the interface shows the muscle activity which corresponds to the coordinates (Max X, Total X, Max Y, and Total Y) along which the heart is pinned out (along the Y axis in this instance (**B**)). Blue columns contain the delta which is the amount of displacement within the given time interval (represented by a bar) detected by the sensor, i.e. the sensor gives no specific coordinates but displacement values. The red line, which changes according to the blue bars, represents the actual position. The delta is always added to the red value. During the phases of the heart contraction the auricle (A) contraction is followed by the ventricle (V) contraction (**C**). A representative auricle and ventricle contraction event recorded by DigiRec is shown on panel **D**.

The graphical interface (Fig. 4) allows the user to start and stop recording, display graphs, and replay the events. The first slider manipulates the width of the graph, while the second one manipulates the resolution in milliseconds. The time is displayed at the bottom along with information about the movements of the heart using the following coordinates: Max X, Total X, Max Y, and Total Y. The first values are movements of the heart in DPI but the software also displays the values in mm. Importantly, in order to display the appropriate mm values, one needs to enter the DPI value of the applied mouse into the “gui.props” utility program. The optical sensor records the changes of the refraction as movements in a positive or negative direction along either axis (Fig. 5).

### Method validation and suitability of the recording system

To assess the suitability of the developed recording system in pharmacologic experiments, we tested if we were able to record a known pharmacological effect on the heart preparations. The substance tested was dopamine which is known to have a cardioexcitatory effect in a variety of molluscan species including *L. stagnalis* [7]. Specifically, dopamine has been shown to increase both heart rate and contraction amplitude in the heart of *L. stagnalis* with slightly increasing the tone level [7].

**Fig. 6:**
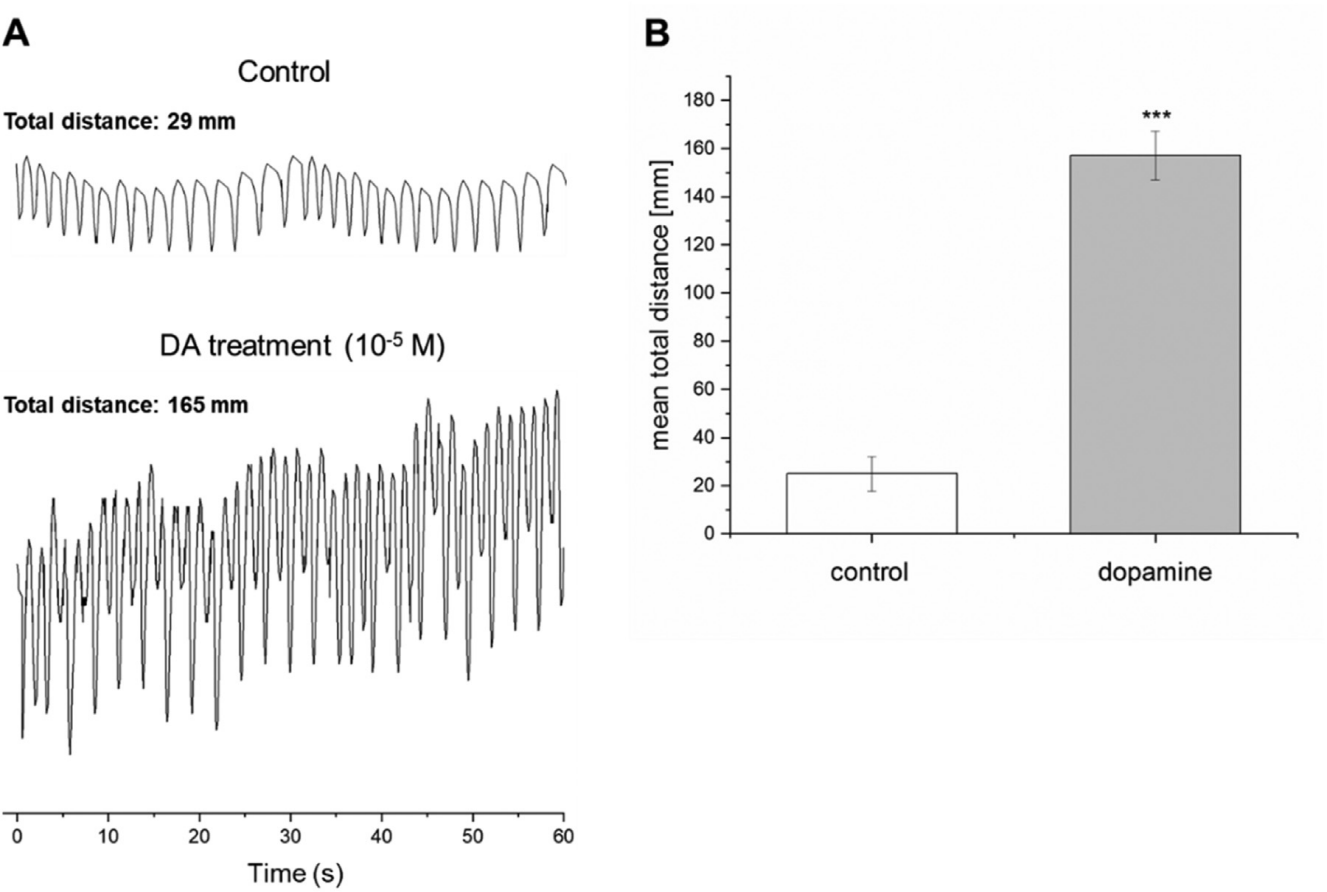
Validation of the developed optical recording system. (**A**) Representative graph illustrating the effects of 10 µM dopamine (DA) on the isolated heart of *L. stagnalis*. The frequency and amplitude have both visibly increased, and the ‘total distance’ measurement has increased. (**B**) Average ‘total distance’ measurements for the control and DA recordings. Each bar represents mean ± SD. The white column shows the control data (before dopamine treatment) while the grey column represents the data after the dopamine treatment. Significance of differences to the control group is marked by asterisks (***P ≤ 0.001, paired t-test).

During the experiments, we tested the effects of a 10 µM dopamine solution on individual heart preparations (n=6). The two parameters investigated, amplitude and frequency, are together referred to as total distance. For each heart preparation, the original heart contractions were recorded as control followed by recording the heart contractions after treatment with dopamine. This always was a continuous recording. Compared to the control recordings, our results demonstrated that dopamine has significantly increased the amplitude and frequency of the contractions (P<0.001; paired t-test) (Fig. 6). Our results confirm dopamine as a cardioactive neurotransmitter in *L. stagnalis* and highlight that the optical recording system is entirely suitable for pharmacological experiments.

### Perspectives

Nowadays, there is a great need to test the physiological effects of different chemicals. The development and optimization of new methods are crucial to move forward such investigations. The refraction-based recording system illustrates the progression of technology from the mechanical force transducer system used not only for *L. stagnalis* research but also for higher organisms. Our system offers a highly suitable tool in ecotoxicology (e.g., testing the effects of pharmacologically active compounds found in the ecosystem) or neuroscience (e.g., testing the effects of different neurotransmitters and neuropeptides).

## Ethics statements

All procedures on animals were performed according to the protocols approved by the Scientific Committee of Animal Experimentation of the Balaton Limnological Research Institute (VE-I-001/01890-10/2013).

## CRediT authorship contribution statement

**Zsolt Pirger:** Conceptualization, Methodology, Investigation, Writing – review & editing, Data curation, Visualization, Supervision, Funding acquisition. **Zita László:** Investigation, Writing – review & editing. **Ildikó Kemenes:** Writing – review & editing. **György Kemenes:** Writing – review & editing. **István Fodor:** Conceptualization, Writing – original draft.

## Declaration of Competing Interest

The authors declare that they have no known competing financial interests or personal relationships that could have appeared to influence the work reported in this paper.

## Data Availability

Data will be made available on request.
